# Pulp Hypertrophy of the Teeth

**Published:** 1903-06

**Authors:** 

**Affiliations:** Strassburg, Germany


### PULP HYPERTROPHY OF THE TEETH.[1]

BY PRIVATDOCENT DR. VON RÖMER, STRASSBURG, GERMANY.

I WAS first led to the histologic examination of pulp hypertrophy by the desire to find the conditions of the nerve-supply in this so highly altered pathologic tissue. In the study of the nerves I found not only what I expected, partly complete absence of nerves

---

[1] Abstract of a paper read before the American Medical Association, Section on Stomatology, New Orleans, May 5 to 8, 1903.

and partly marked decrease of nerve-fibres, thus explaining the great difference of sensitiveness, but also some other very interesting discoveries which I believe are new.

Pulp hypertrophy is to be considered as a new tissue formation due to chronic pulpitis from exposure through more or less complete destruction of the overlying protective tissues. This new growth consists of a comparatively coarse granulation tissue in which three layers can be distinguished. The outer layer consists of a thick stratum of white blood- or pus-corpuscles beginning to break down, then follows a somewhat wider zone consisting of a proliferation of endothelial cells and capillary vessels, which, seen with a less magnifying power, has almost the appearance of granular layer, similar to that before described in the so-called endothelial granuloma of the dental root. The third layer, which forms the chief mass of the tumor, consists of a coarse connective tissue with dilated blood-vessels interspersed with crowded round cells.

Up to the present I have studied about thirty different specimens, mostly obtained in clinics, but for part of which I have to thank my colleagues. I have prepared them in the following manner: First, the whole tooth with the pulp *in situ* in a ten per cent. formalin solution, then decalcified it in a thirty-three and one-third per cent. formic acid solution (which is, as a rule, accomplished in two or three weeks), then divided the tooth, together with the hypertrophied pulp, into two nearly equal halves. Of the halves, such as seemed most suited for further treatment for microscopic examination was taken. These were embedded in celloidin and cut with the microtome into the thinnest possible layers and then stained with alum, hæmatoxylin, and picrofuchsin (Van Gieson), and part with Weigert's medullary staining process, in which the nerves and their medullary layers appear as dark, blue-colored fibres on a yellowish background. This shows that in so-called pulp hypertrophy (which arises like a fungus from the pulp-chamber and generally fills the carious part crown of the tooth) nerve-fibres are absent. In the pulp-cavity itself and in the root-canals, on the other hand, numerous nerve-bundles are found according to the degree of degeneration of the pulp-tissue. This degeneration consists in a transformation into granulation tissue, particularly where infiltration with round cells changes into granulation. The nerve-fibres are already broken down or beginning to be degenerated, as shown by distortions, interruptions, and knotformed swellings of the nerve-fibres. Such degenerative processes of the nerves can best be seen in pulps which have been laid bare by fracture in attempts at extraction, and in which the superficies of the exposed pulp is changed into granulation tissue and pulp hypertrophy.

The study of preparations stained by the Van Gieson method has convinced me that the difference claimed by Rothmund and Arkövy between pulp hypertrophy and pulpitis chronica hypertrophica granulomatosa does not exist. By the pulpitis chronica hypertrophica granulomatosa is understood (according to Rothmund and Arkövy; compare Scheff's Handbuch, Band ii. Seite 297) a granulating mass of the pulp from the size of a millet-seed to a pea, soft, with little consistency, and extending over one or two pulp-points. The hypertrophy, on the other hand, which Arkövy calls pulpitis chronica sarcomatosa is a compact, dense neoplasm caused by proliferation of the pulp-tissue, distinctly limited, generally smooth, and only here and there slightly lobular, less vascular, extending over the whole pulp-cavity, except perhaps some very slight portion including the whole crown cavity of the tooth.

I find neither clinically nor histologically any noticeable difference in the two forms. Both forms of hypertrophic pulpitis differ only in their size, and agree perfectly in their histologic structure. The fleshy, round bean or hazel-nut pulp hypertrophy develops simply through its natural growth from the minute millet-seed or pea-sized neoplasm. In both cases there is a degeneration of pulp-tissue and transformation into a more or less coarse granulation tissue, which consists of connective tissue fibres with numerous blood-vessels, round cells, and proliferated endothelium and connective-tissue cells.

The striking point in all the specimens is the richness of blood-supply. The vessels are not only numerous, but also dilated, almost like a hæmangeioma, so that after extraction of the tooth the hypertrophy loses half its volume.

In almost all cases of pulp hypertrophy there is found to some extent the formation of partly attached, partly free pulp-stones and calcareous deposits. This is in itself not astonishing, since in nearly all teeth which suffer from any kind of pulpitis, calcic formations are present to the discomfiture of the investigator, and even in well-decalcified preparations they often cause inconvenient nicking of the microtome knife. But what is here remarkable is the fact that in many hypertrophied pulps such calcareous formations occur well above the pulp-chamber and the hypertrophy itself. These calcareous masses or pulp-stones are in part round or oval, with distinct stratifications and partly irregularly dentate, and show cell inclusions similar to those which are found in the formation of secondary dentine inside the pulp-chamber.

How can these things be explained? I believe the pulp hypertrophy only occurs in teeth, the pulp elements of which possess great vital energy and capacity for regeneration and in which they can oppose special resistance against incoming bacteria and other morbid influences.

The formation of secondary dentine partly attached to the walls and partly free is to be considered an attempt at defence from external injury. The quicker the pulp is compelled to form secondary dentine, the more irregular will be its formation, so the calcification does not follow regular rules and the dentine is not laid down in regular layers, but the pulp-cells are included in rapidly formed irregular dentine structure. Such pulp-cells, so over-energetic in dentine formation, may, here and there, extend with the granulation tissue beyond the pulp-chamber itself, and thus, far away from their original site, form on the crown of the hypertrophy such pulp-stones as are shown in some of my specimens.

Some things shown appear at first rather difficult to explain. Thus, in one case the surface is covered with regular stratified epithelium instead of the usual round cells and pus-corpuscles, also with the high papillæ which are typical of the gum tissue. How does this epithelium develop on the pulp? Usually the excised fragment of a pulp hypertrophy is readily distinguished from a corresponding fragment of an alveolar growth by the absence of epithelium. I explain the process as follows: Hypertrophied pulps are frequently ingrafted with alveolar growths; that is, when a pulp hypertrophy extends outside a pulp-chamber over the corroded wall of the crown in contact with the gum tissue. Both are easily injured in chewing, so that spontaneous auto-transplantation from the gum tissue to the pulp hypertrophy occurs. Six months after preparing the one specimen I found another which demonstrated conclusively the truth of this conjecture. I have seen gum tissue grown over the completely destroyed crown of the tooth of which one was united with the broad surface of the pulp hypertrophy in the pulp-chamber. The whole surface of this hypertrophy was already covered with epithelium of the same composition and height of papillæ as that possessed by the alveolar tissue. Later I had the opportunity to separate the gum overgrowth from the pulp and there remained then only the pulp hypertrophy covered with epithelium.

In conclusion, a few words on the therapy of pulp hypertrophies. In cases where the pulp has one of these growths (they are vascular and at the same time poorly innervated), they may be either simply amputated with a pair of fine scissors or pointed knife in the depth of the pulp-chamber and then arsenic paste applied to cause necrosis of the root-pulp, or the arsenic paste can be applied direct to the crown of the hypertrophy in order to destroy by necrosis the entire tissue. But there is no serious error, in my opinion, in avoiding the use of arsenic altogether, but simply cut off the top of the hypertrophy and cap the stump.

[REMARKS.—If the author of this interesting paper will refer to page 857, "American System of Dentistry," he will find there that Dr. G. V. Black gave, in 1886, much of the supposed "new" views embodied in the foregoing article.—ED.]

